# Andirobin from *X. moluccensis*

**DOI:** 10.1107/S1600536812027705

**Published:** 2012-07-25

**Authors:** Chutima Jittaniyom, Damrong Sommit, Nongnuj Muangsin, Khanitha Pudhom

**Affiliations:** a Department of Chemistry, Faculty of Science, Chulalongkorn University, Bangkok 10330, Thailand; b Department of Chemistry, Faculty of Science, Mahanakorn University of Technology, Bangkok 10530, Thailand; c Research Centre of Bioorganic Chemistry, Department of Chemistry, Faculty of Science, Chulalongkorn University, Bangkok, 10330, Thailand; d Center for Petroleum, Petrochemicals, and Advanced Materials, Chulalongkorn University, Bangkok 10330, Thailand

## Abstract

The title compound (systematic name: methyl 2-{(1*R*,2*R*)-2-[(1a*S*,4*S*,4a*S*,8a*S*)-4-(furan-3-yl)-4a-methyl-8-methyl­ene-2-oxoocta­hydro­oxireno[2,3-*d*]isochromen-7-yl]-2,6,6-trimethyl-5-oxocyclo­hex-3-en-1-yl}acetate), C_27_H_32_O_7_, was isolated from *X. moluccensis* seeds from Thailand. The conformations of the six-membered rings are distorted half-chair, chair and half-chair for the isolated cyclo­hexane, fused cyclo­hexane and lactone rings, respectively. In addition, the lactone ring bears in an equatorial orientation an essentially planar furan ring (r.m.s. deviation = 0.004 Å), which forms an angle of 63.87 (13)° with the mean plane defined by the ten atoms of the two fused six-membered rings (r.m.s. deviation = 0.213 Å). The absolute configuration was fixed on the basis of literature data.

## Related literature

For general background to limonoids and their activities, see: Alvi *et al.* (1991 ▶); Yu *et al.* (2007 ▶); Li *et al.* (2009 ▶). For related structures, see: Chanin *et al.* (2010 ▶); Pudhom *et al.* (2009 ▶, 2010 ▶). For the bioactivity of limonoids, see: Koul *et al.* (2004 ▶); Endo *et al.* (2002 ▶); Nakagawa *et al.* (2001 ▶); Ravangpai *et al.* (2011 ▶). For puckering parameters, see: Cremer & Pople (1975 ▶).
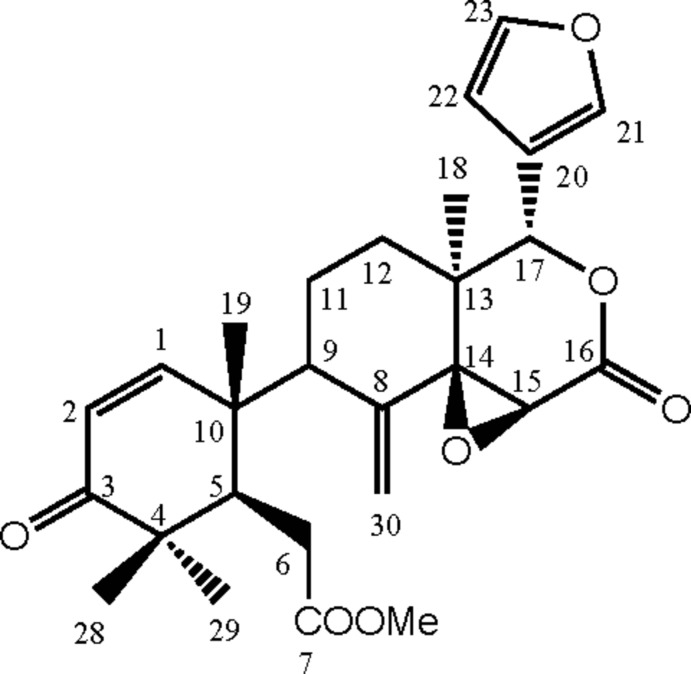


## Experimental

### 

#### Crystal data

C_27_H_32_O_7_
*M* *_r_* = 468.53
Orthorhombic, 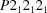

*a* = 8.8125 (5) Å
*b* = 12.5907 (7) Å
*c* = 21.9393 (11) Å
*V* = 2434.3 (2) Å^3^
*Z* = 4
Mo *K*α radiation
μ = 0.09 mm^−1^
*T* = 296 K
0.48 × 0.40 × 0.36 mm

#### Data collection

Bruker SMART APEXII CCD area-detector diffractometer
13719 measured reflections
3132 independent reflections
2725 reflections with *I* > 2σ(*I*)
*R*_int_ = 0.020

#### Refinement

*R*[*F* ^2^ > 2σ(*F* ^2^)] = 0.041
*wR*(*F* ^2^) = 0.117
*S* = 1.11
5520 reflections
312 parameters
H-atom parameters constrained
Δρ_max_ = 0.68 e Å^−3^
Δρ_min_ = −0.18 e Å^−3^

### 

Data collection: *APEX2* (Bruker, 2008 ▶); cell refinement: *SAINT* (Bruker, 2008 ▶); data reduction: *SAINT*; program(s) used to solve structure: *SHELXS97* (Sheldrick, 2008 ▶); program(s) used to refine structure: *SHELXL97* (Sheldrick, 2008 ▶); molecular graphics: *ORTEP-3* (Farrugia, 1997 ▶; software used to prepare material for publication: *publCIF* (Westrip, 2010 ▶).

## Supplementary Material

Crystal structure: contains datablock(s) global, I. DOI: 10.1107/S1600536812027705/lr2061sup1.cif

Structure factors: contains datablock(s) I. DOI: 10.1107/S1600536812027705/lr2061Isup2.hkl

Supplementary material file. DOI: 10.1107/S1600536812027705/lr2061Isup3.cml

Additional supplementary materials:  crystallographic information; 3D view; checkCIF report

## Figures and Tables

**Fig. 1.**
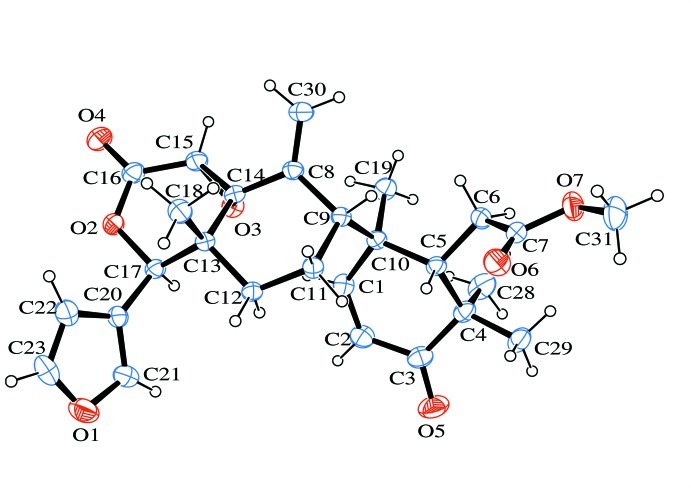
The molecular structure of the title compound with ellipsoids drawn at the 30% probability level.

## supplementary crystallographic information

### Comment

Limonoids are triterpene derivatives from a precursor with a 4,4,8-trimethyl-17-furanylsteroid skeleton. Limonoid examination of the Meliaceae family is of growing interest due to a range of biological activities, such as insect antifeedants and growth regulators, antibacterial, antifungal, antimalarial, anticancer, antiviral and anti-inflammatory activities (Koul *et al.*, 2004; Endo *et al.*, 2002; Nakagawa *et al.*, 2001; Ravangpai, *et al.*, 2011). The genus *Xylocarpus* (Meliaceae) has proved to be a rich source of an array of structurally diverse limonoids, including gedunin, andirobin, mexicanolide and phragmalin type limonoids, with a broad range of biological activities (Alvi *et al.*, 1991; Yu *et al.*, 2007; Li *et al.*, 2009). We have recently reported the isolation and identification a number of limonoids from three Thai mangroves in this genus, *X. granatum*, *X. moluccensis* and *X. rumphii* (Chanin *et al.*, 2010; Pudhom *et al.*, 2009; Pudhom *et al.*, 2010). Herein, we report the complete assignments of NMR and the crystal structure of the title compound isolated from *X. moluccensis* seeds.

In the molecular structure, the conformation of the six-membered rings are distorted half-chair, chair and half-chair for the isolated cyclohenane, fused cyclohexane and lactone ring respectively (Cremer & Pople, 1975). In addition, the lactone ring bears in equatorial orientation a planar furan ring (r.m.s. deviation= 0.004 Å) which form an angle of 63.87 (13)^o ^with the mean square plane (r.m.s. deviation Å) defined by the ten atoms of the two fused six-membered rings.

### Experimental

General Experiment Procedures. Melting point was measured using a Fisher-Johns melting point apparatus. NMR spectra were recorded with a Bruker AV400 (^1^H, 400 MHz; ^13^C, 100 MHz) spectrometer using tetramethylsilane as an internal standard. Mass spectra were obtained from a Bruker micrOTOF mass spectrometer.

Plant Material. Fruits of *X. moluccensis* were collected from Surat Thani province, Thailand, in January 2010. Plant materials were identified by Royal Forest Department, Bangkok, Thailand.

Extraction and Isolation of Andirobin (1). Air-dried powdered seeds of *X. moluccensis* (2 kg) were extracted with MeOH (5*L x* 2, each for two days) at room temperature. Extracts were pooled and the solvent were removed under reduced pressure. The combined MeOH extract was then suspended in water and partitioned with EtOAc. The EtOAc crude extract obtained (30 g) was chromatographed on a sililca gel column eluted with a gradient of acetone-hexane (from 1:9 to 1:0) to yield 12 fractions. Fraction 2 was further purified by silica gel column chromatography eluting with a 1:9 mixture of acetone-hexane and recrystallized from MeOH to afford the title compound (1, 25.0 mg).

Andirobin (1): colorless crystals; ^1^H NMR (400 MHz, CDCl_3_) *d* 7.34 (1*H*, s, H-23), 7.33 (1*H*, s, H-21), 7.07 (1*H*, d, *J* = 10.4 Hz, H-1), 6.27 (1*H*, s, H-22), 5.99 (1*H*, d, *J* = 10.4 Hz, H-2), 5.41 (1*H*, s, H-17), 5.30 (1*H*, s, H-30*a*), 5.20 (1*H*, s, H-30*b*), 3.97 (1*H*, s, H-15), 3.64 (3*H*, s, 7-COO*CH_3_*), 2.62 (1*H*, dd, *J* = 3.2, 6.8 Hz, H-5), 2.44 (1*H*, dd, *J* = 7.2, 17.2 Hz, H-6a), 2.39 (1*H*, d, *J* = 6.8 Hz, H-9), 2.28 (1*H*, dd, *J* = 3.2, 17.2 Hz, H-6 b), 1.90 (1*H*, m, H-11*a*), 1.73 (1*H*, m, H-11*b*), 1.60 (1*H*, m, H-12*a*), 1.16 (1*H*, m, H-12*b*), 1.04 (3*H*, s, 28-CH_3_), 1.01 (3*H*, s, 29-CH_3_), 0.90 (3*H*, s, 19-CH_3_), 0.87 (3*H*, s, 18-CH_3_);

^13^C NMR (100 MHz, CDCl_3_) *d* 203.7 (C=O, C-3), 174.3 (C=O, C-7), 166.7 (C=O, C-16), 153.5 (CH, C-1), 143.2 (CH, C-23), 140.9 (CH, C-21), 138.9 (C, C-8), 125.7 (CH, C-2), 122.3 (CH_2_, C-30), 119.8 (C, C-20), 109.7 (CH, C-22), 77.4 (CH, C-15), 67.8 (C, C-14), 55.5 (CH, C-17), 52.1 (CH_3_, 7-COO*CH_3_*), 48.8 (CH, C-9), 46.1 (C, C-4), 43.1 (C, C-10), 42.8 (CH, C-5), 38.6 (C, C-13), 31.5 (CH_2_, C-6), 29.5 (CH_2_, C-12), 22.7 (CH_3_, C-29), 22.5 (CH_3_, C-28), 21.3 (CH_2_, C-11), 20.2 (CH_3_, C-19), 14.6 (CH_3_, C-18).

### Refinement

All H atoms were geometrically positioned and treated as riding atoms with distances C—H = 0.96 Å (CH3), 0.97 Å (CH2), 0.93 Å (CH), and *U*_iso_(H) = 1.20 *U*_eq_(C) for methylene and aromatic, 1.50 *U*_eq_(C) for methyl. The absolute structure could not be determined from the X-ray analysis, but it was known from earlier work on related compounds (*e.g.* Alvi *et al.*, 1991, Yu *et al.*, 2007 and Li *et al.*, 2009). 2388 Friedel pairs were therefore merged before the final refinement.

The maximum residual density ( 0.68 eÅ^3^) is larger than normally expected. However, the nearest atom to the corresponding minimum is O5 at 2.74 Å, which seems to indicate that de residual density can be associated to unmodeled disordered solvent molecules.

### Crystal data

**Table:**

C_27_H_32_O_7_	*Z* = 4
*M**_r_* = 468.53	*F*(000) = 1000
Orthorhombic, *P*2_1_2_1_2_1_	*D*_x_ = 1.278 Mg m^−^^3^
Hall symbol: P 2ac 2ab	Mo *K*α radiation, λ = 0.71073 Å
*a* = 8.8125 (5) Å	µ = 0.09 mm^−^^1^
*b* = 12.5907 (7) Å	*T* = 296 K
*c* = 21.9393 (11) Å	Prism, colourless
*V* = 2434.3 (2) Å^3^	0.48 × 0.40 × 0.36 mm

### Data collection

**Table:**

Bruker SMART APEXII CCD area-detector diffractometer	2725 reflections with *I* > 2σ(*I*)
Radiation source: Mo Kα	*R*_int_ = 0.020
Graphite monochromator	θ_max_ = 27.5°, θ_min_ = 1.9°
φ and ω scans	*h* = −11→7
13719 measured reflections	*k* = −15→15
3132 independent reflections	*l* = −28→19

### Refinement

**Table:**

Refinement on *F*^2^	0 restraints
Least-squares matrix: full	H-atom parameters constrained
*R*[*F*^2^ > 2σ(*F*^2^)] = 0.041	*w* = 1/[σ^2^(*F*_o_^2^) + (0.0714*P*)^2^ + 0.1995*P*] where *P* = (*F*_o_^2^ + 2*F*_c_^2^)/3
*wR*(*F*^2^) = 0.117	(Δ/σ)_max_ = 0.014
*S* = 1.11	Δρ_max_ = 0.68 e Å^−^^3^
5520 reflections	Δρ_min_ = −0.18 e Å^−^^3^
312 parameters	

### Special details

**Table:**

Geometry. All e.s.d.'s (except the e.s.d. in the dihedral angle between two l.s. planes) are estimated using the full covariance matrix. The cell e.s.d.'s are taken into account individually in the estimation of e.s.d.'s in distances, angles and torsion angles; correlations between e.s.d.'s in cell parameters are only used when they are defined by crystal symmetry. An approximate (isotropic) treatment of cell e.s.d.'s is used for estimating e.s.d.'s involving l.s. planes.

### Fractional atomic coordinates and isotropic or equivalent isotropic displacement parameters (Å^2^)

**Table:**

	*x*	*y*	*z*	*U*_iso_*/*U*_eq_	
C1	0.8749 (3)	0.19440 (18)	0.08892 (10)	0.0410 (5)	
H1	0.7868	0.1870	0.0660	0.049*	
C2	0.9926 (3)	0.2397 (2)	0.06213 (12)	0.0504 (6)	
H2	0.9842	0.2570	0.0211	0.060*	
C3	1.1348 (3)	0.2640 (2)	0.09316 (12)	0.0487 (6)	
C4	1.1303 (3)	0.25998 (19)	0.16248 (12)	0.0427 (5)	
C5	1.0339 (2)	0.16189 (17)	0.18228 (10)	0.0344 (4)	
H5	1.0890	0.1003	0.1664	0.041*	
C6	1.0335 (3)	0.1469 (2)	0.25150 (11)	0.0417 (5)	
H6A	1.0424	0.2159	0.2708	0.050*	
H6B	0.9369	0.1166	0.2636	0.050*	
C7	1.1586 (3)	0.07683 (19)	0.27427 (10)	0.0406 (5)	
C8	0.6587 (2)	0.00557 (17)	0.15184 (10)	0.0352 (4)	
C9	0.8277 (2)	0.03182 (16)	0.15559 (9)	0.0324 (4)	
H9	0.8598	0.0073	0.1960	0.039*	
C10	0.8716 (2)	0.15387 (17)	0.15342 (10)	0.0338 (4)	
C11	0.9130 (2)	−0.04026 (19)	0.10972 (10)	0.0377 (5)	
H11A	1.0170	−0.0156	0.1068	0.045*	
H11B	0.9156	−0.1118	0.1261	0.045*	
C12	0.8468 (2)	−0.04512 (19)	0.04580 (10)	0.0364 (4)	
H12A	0.9024	−0.0975	0.0223	0.044*	
H12B	0.8606	0.0233	0.0262	0.044*	
C13	0.6761 (2)	−0.07398 (16)	0.04482 (9)	0.0321 (4)	
C14	0.5934 (2)	−0.00153 (17)	0.08910 (10)	0.0338 (4)	
C15	0.4320 (2)	0.0194 (2)	0.07674 (11)	0.0436 (5)	
H15	0.3670	0.0315	0.1123	0.052*	
C16	0.3595 (3)	−0.0313 (2)	0.02300 (11)	0.0466 (6)	
C17	0.6142 (2)	−0.05036 (19)	−0.01938 (10)	0.0369 (5)	
H17	0.6351	0.0243	−0.0288	0.044*	
C18	0.6499 (3)	−0.18975 (18)	0.06402 (11)	0.0447 (5)	
H18A	0.5447	−0.2074	0.0588	0.067*	
H18B	0.6776	−0.1984	0.1060	0.067*	
H18C	0.7109	−0.2358	0.0392	0.067*	
C19	0.7500 (3)	0.21722 (19)	0.18871 (12)	0.0447 (6)	
H19A	0.7865	0.2878	0.1964	0.067*	
H19B	0.7291	0.1825	0.2267	0.067*	
H19C	0.6587	0.2208	0.1649	0.067*	
C20	0.6791 (3)	−0.11713 (19)	−0.06933 (10)	0.0394 (5)	
C21	0.7912 (3)	−0.0869 (2)	−0.10764 (12)	0.0569 (7)	
H21	0.8380	−0.0207	−0.1070	0.068*	
C22	0.6448 (4)	−0.2222 (2)	−0.08653 (13)	0.0603 (7)	
H22	0.5727	−0.2659	−0.0684	0.072*	
C23	0.7334 (4)	−0.2479 (3)	−0.13315 (12)	0.0614 (8)	
H23	0.7318	−0.3129	−0.1533	0.074*	
C28	1.0646 (3)	0.3679 (2)	0.18309 (17)	0.0640 (8)	
H28A	1.0457	0.3662	0.2262	0.096*	
H28B	0.9713	0.3813	0.1618	0.096*	
H28C	1.1360	0.4233	0.1741	0.096*	
C29	1.2920 (3)	0.2503 (2)	0.18786 (14)	0.0534 (6)	
H29A	1.3322	0.1816	0.1780	0.080*	
H29B	1.2898	0.2590	0.2313	0.080*	
H29C	1.3549	0.3043	0.1701	0.080*	
C30	0.5775 (3)	−0.0189 (2)	0.20065 (11)	0.0503 (6)	
H30A	0.4774	−0.0411	0.1963	0.060*	
H30B	0.6205	−0.0139	0.2392	0.060*	
C31	1.3128 (4)	0.0371 (3)	0.35953 (15)	0.0744 (9)	
H31A	1.2729	−0.0330	0.3658	0.112*	
H31B	1.3411	0.0674	0.3980	0.112*	
H31C	1.4004	0.0333	0.3336	0.112*	
O1	0.8261 (3)	−0.1661 (2)	−0.14718 (10)	0.0712 (6)	
O2	0.44932 (17)	−0.06685 (16)	−0.02238 (8)	0.0484 (4)	
O3	0.54407 (18)	0.09958 (13)	0.06392 (8)	0.0427 (4)	
O4	0.22495 (19)	−0.0432 (2)	0.01901 (10)	0.0681 (6)	
O5	1.2467 (2)	0.2941 (2)	0.06570 (11)	0.0795 (7)	
O6	1.2129 (2)	0.00409 (15)	0.24700 (8)	0.0535 (4)	
O7	1.1985 (2)	0.10284 (18)	0.33109 (8)	0.0591 (5)	

### Atomic displacement parameters (Å^2^)

**Table:**

	*U*^11^	*U*^22^	*U*^33^	*U*^12^	*U*^13^	*U*^23^
C1	0.0448 (12)	0.0358 (11)	0.0423 (12)	0.0015 (10)	−0.0057 (10)	0.0053 (9)
C2	0.0575 (14)	0.0491 (14)	0.0446 (13)	−0.0022 (12)	0.0010 (11)	0.0135 (11)
C3	0.0472 (13)	0.0420 (12)	0.0571 (15)	−0.0019 (11)	0.0068 (12)	0.0113 (11)
C4	0.0369 (10)	0.0364 (11)	0.0549 (14)	−0.0032 (9)	0.0027 (10)	−0.0047 (10)
C5	0.0313 (9)	0.0342 (10)	0.0377 (11)	0.0007 (9)	0.0029 (9)	−0.0039 (9)
C6	0.0383 (11)	0.0486 (13)	0.0380 (11)	0.0017 (10)	0.0011 (10)	−0.0083 (10)
C7	0.0340 (10)	0.0505 (13)	0.0373 (11)	−0.0068 (10)	0.0002 (9)	−0.0020 (10)
C8	0.0333 (9)	0.0357 (10)	0.0368 (10)	0.0013 (9)	0.0054 (9)	−0.0015 (9)
C9	0.0317 (9)	0.0339 (10)	0.0316 (9)	0.0027 (8)	0.0000 (8)	0.0026 (8)
C10	0.0317 (9)	0.0330 (10)	0.0368 (10)	0.0028 (8)	0.0011 (9)	−0.0006 (8)
C11	0.0320 (9)	0.0398 (11)	0.0414 (11)	0.0074 (9)	−0.0018 (9)	−0.0077 (10)
C12	0.0293 (9)	0.0411 (11)	0.0386 (10)	0.0025 (9)	0.0041 (9)	−0.0052 (9)
C13	0.0306 (9)	0.0333 (10)	0.0325 (10)	−0.0015 (8)	0.0019 (8)	0.0001 (8)
C14	0.0295 (9)	0.0351 (10)	0.0367 (10)	0.0001 (8)	0.0056 (8)	0.0020 (9)
C15	0.0309 (10)	0.0559 (14)	0.0438 (12)	0.0045 (10)	0.0045 (9)	−0.0038 (11)
C16	0.0320 (10)	0.0609 (15)	0.0470 (13)	0.0008 (11)	0.0005 (10)	0.0015 (11)
C17	0.0299 (9)	0.0444 (11)	0.0365 (10)	−0.0002 (9)	0.0006 (8)	0.0017 (10)
C18	0.0554 (13)	0.0355 (11)	0.0433 (12)	−0.0041 (11)	0.0017 (11)	0.0028 (9)
C19	0.0380 (11)	0.0396 (12)	0.0565 (14)	0.0083 (10)	0.0023 (11)	−0.0089 (11)
C20	0.0365 (10)	0.0490 (12)	0.0327 (10)	−0.0007 (10)	−0.0034 (9)	0.0000 (10)
C21	0.0575 (15)	0.0646 (16)	0.0486 (14)	−0.0055 (14)	0.0143 (13)	−0.0092 (13)
C22	0.0700 (18)	0.0625 (16)	0.0485 (14)	−0.0169 (15)	0.0043 (14)	−0.0103 (13)
C23	0.079 (2)	0.0610 (17)	0.0440 (14)	0.0081 (16)	−0.0053 (14)	−0.0161 (13)
C28	0.0568 (16)	0.0386 (13)	0.097 (2)	−0.0021 (12)	0.0090 (16)	−0.0122 (14)
C29	0.0393 (11)	0.0538 (15)	0.0671 (16)	−0.0080 (12)	−0.0017 (12)	−0.0075 (13)
C30	0.0443 (12)	0.0640 (16)	0.0426 (12)	−0.0058 (12)	0.0092 (11)	0.0043 (12)
C31	0.0599 (16)	0.102 (3)	0.0610 (17)	0.0075 (19)	−0.0247 (15)	−0.0005 (18)
O1	0.0636 (12)	0.0953 (17)	0.0548 (11)	0.0116 (13)	0.0136 (10)	−0.0144 (11)
O2	0.0303 (7)	0.0707 (12)	0.0441 (9)	−0.0009 (8)	−0.0025 (7)	−0.0084 (8)
O3	0.0379 (7)	0.0408 (8)	0.0493 (9)	0.0077 (7)	−0.0028 (7)	0.0008 (7)
O4	0.0308 (8)	0.1065 (17)	0.0671 (12)	−0.0061 (10)	0.0017 (9)	−0.0101 (13)
O5	0.0559 (12)	0.110 (2)	0.0727 (13)	−0.0154 (12)	0.0136 (11)	0.0291 (14)
O6	0.0519 (10)	0.0581 (11)	0.0504 (10)	0.0105 (9)	−0.0017 (8)	−0.0012 (9)
O7	0.0537 (10)	0.0788 (13)	0.0450 (10)	0.0036 (10)	−0.0149 (8)	−0.0079 (9)

### Geometric parameters (Å, º)

**Table:**

C1—C2	1.321 (3)	C14—C15	1.471 (3)
C1—C10	1.505 (3)	C15—O3	1.440 (3)
C1—H1	0.9300	C15—C16	1.485 (3)
C2—C3	1.459 (4)	C15—H15	0.9800
C2—H2	0.9300	C16—O4	1.198 (3)
C3—O5	1.215 (3)	C16—O2	1.348 (3)
C3—C4	1.522 (4)	C17—O2	1.469 (2)
C4—C29	1.534 (3)	C17—C20	1.495 (3)
C4—C28	1.545 (3)	C17—H17	0.9800
C4—C5	1.561 (3)	C18—H18A	0.9600
C5—C6	1.530 (3)	C18—H18B	0.9600
C5—C10	1.567 (3)	C18—H18C	0.9600
C5—H5	0.9800	C19—H19A	0.9600
C6—C7	1.498 (3)	C19—H19B	0.9600
C6—H6A	0.9700	C19—H19C	0.9600
C6—H6B	0.9700	C20—C21	1.351 (4)
C7—O6	1.194 (3)	C20—C22	1.408 (4)
C7—O7	1.336 (3)	C21—O1	1.358 (3)
C8—C30	1.324 (3)	C21—H21	0.9300
C8—C14	1.495 (3)	C22—C23	1.327 (4)
C8—C9	1.528 (3)	C22—H22	0.9300
C9—C11	1.550 (3)	C23—O1	1.350 (4)
C9—C10	1.585 (3)	C23—H23	0.9300
C9—H9	0.9800	C28—H28A	0.9600
C10—C19	1.545 (3)	C28—H28B	0.9600
C11—C12	1.520 (3)	C28—H28C	0.9600
C11—H11A	0.9700	C29—H29A	0.9600
C11—H11B	0.9700	C29—H29B	0.9600
C12—C13	1.547 (3)	C29—H29C	0.9600
C12—H12A	0.9700	C30—H30A	0.9300
C12—H12B	0.9700	C30—H30B	0.9300
C13—C14	1.519 (3)	C31—O7	1.445 (4)
C13—C18	1.535 (3)	C31—H31A	0.9600
C13—C17	1.540 (3)	C31—H31B	0.9600
C14—O3	1.454 (3)	C31—H31C	0.9600
			
C2—C1—C10	125.4 (2)	C15—C14—C13	116.97 (19)
C2—C1—H1	117.3	C8—C14—C13	116.10 (17)
C10—C1—H1	117.3	O3—C15—C14	59.93 (13)
C1—C2—C3	123.9 (2)	O3—C15—C16	116.2 (2)
C1—C2—H2	118.1	C14—C15—C16	119.0 (2)
C3—C2—H2	118.1	O3—C15—H15	116.5
O5—C3—C2	122.1 (2)	C14—C15—H15	116.5
O5—C3—C4	121.8 (3)	C16—C15—H15	116.5
C2—C3—C4	115.9 (2)	O4—C16—O2	119.0 (2)
C3—C4—C29	109.9 (2)	O4—C16—C15	122.5 (2)
C3—C4—C28	105.8 (2)	O2—C16—C15	118.41 (18)
C29—C4—C28	108.2 (2)	O2—C17—C20	105.46 (18)
C3—C4—C5	108.59 (19)	O2—C17—C13	111.37 (17)
C29—C4—C5	110.0 (2)	C20—C17—C13	115.22 (18)
C28—C4—C5	114.23 (19)	O2—C17—H17	108.2
C6—C5—C4	112.02 (19)	C20—C17—H17	108.2
C6—C5—C10	113.03 (17)	C13—C17—H17	108.2
C4—C5—C10	115.81 (18)	C13—C18—H18A	109.5
C6—C5—H5	104.9	C13—C18—H18B	109.5
C4—C5—H5	104.9	H18A—C18—H18B	109.5
C10—C5—H5	104.9	C13—C18—H18C	109.5
C7—C6—C5	113.70 (19)	H18A—C18—H18C	109.5
C7—C6—H6A	108.8	H18B—C18—H18C	109.5
C5—C6—H6A	108.8	C10—C19—H19A	109.5
C7—C6—H6B	108.8	C10—C19—H19B	109.5
C5—C6—H6B	108.8	H19A—C19—H19B	109.5
H6A—C6—H6B	107.7	C10—C19—H19C	109.5
O6—C7—O7	123.4 (2)	H19A—C19—H19C	109.5
O6—C7—C6	125.4 (2)	H19B—C19—H19C	109.5
O7—C7—C6	111.1 (2)	C21—C20—C22	104.8 (2)
C30—C8—C14	121.5 (2)	C21—C20—C17	125.2 (2)
C30—C8—C9	122.2 (2)	C22—C20—C17	130.0 (2)
C14—C8—C9	115.96 (17)	C20—C21—O1	110.9 (3)
C8—C9—C11	108.14 (17)	C20—C21—H21	124.6
C8—C9—C10	116.50 (17)	O1—C21—H21	124.6
C11—C9—C10	115.45 (17)	C23—C22—C20	108.0 (3)
C8—C9—H9	105.2	C23—C22—H22	126.0
C11—C9—H9	105.2	C20—C22—H22	126.0
C10—C9—H9	105.2	C22—C23—O1	110.2 (3)
C1—C10—C19	108.03 (19)	C22—C23—H23	124.9
C1—C10—C5	109.93 (18)	O1—C23—H23	124.9
C19—C10—C5	113.42 (18)	C4—C28—H28A	109.5
C1—C10—C9	111.20 (17)	C4—C28—H28B	109.5
C19—C10—C9	108.42 (17)	H28A—C28—H28B	109.5
C5—C10—C9	105.86 (16)	C4—C28—H28C	109.5
C12—C11—C9	115.89 (17)	H28A—C28—H28C	109.5
C12—C11—H11A	108.3	H28B—C28—H28C	109.5
C9—C11—H11A	108.3	C4—C29—H29A	109.5
C12—C11—H11B	108.3	C4—C29—H29B	109.5
C9—C11—H11B	108.3	H29A—C29—H29B	109.5
H11A—C11—H11B	107.4	C4—C29—H29C	109.5
C11—C12—C13	113.28 (18)	H29A—C29—H29C	109.5
C11—C12—H12A	108.9	H29B—C29—H29C	109.5
C13—C12—H12A	108.9	C8—C30—H30A	120.0
C11—C12—H12B	108.9	C8—C30—H30B	120.0
C13—C12—H12B	108.9	H30A—C30—H30B	120.0
H12A—C12—H12B	107.7	O7—C31—H31A	109.5
C14—C13—C18	108.80 (18)	O7—C31—H31B	109.5
C14—C13—C17	107.38 (17)	H31A—C31—H31B	109.5
C18—C13—C17	112.39 (18)	O7—C31—H31C	109.5
C14—C13—C12	108.47 (17)	H31A—C31—H31C	109.5
C18—C13—C12	111.46 (19)	H31B—C31—H31C	109.5
C17—C13—C12	108.19 (17)	C23—O1—C21	106.1 (2)
O3—C14—C15	58.98 (15)	C16—O2—C17	120.06 (18)
O3—C14—C8	114.37 (18)	C15—O3—C14	61.09 (14)
C15—C14—C8	122.08 (19)	C7—O7—C31	116.5 (2)
O3—C14—C13	115.22 (17)		
			
C10—C1—C2—C3	−4.6 (4)	C9—C8—C14—C15	−153.4 (2)
C1—C2—C3—O5	171.1 (3)	C30—C8—C14—C13	−122.1 (2)
C1—C2—C3—C4	−14.6 (4)	C9—C8—C14—C13	52.1 (3)
O5—C3—C4—C29	−24.3 (4)	C18—C13—C14—O3	−151.59 (18)
C2—C3—C4—C29	161.4 (2)	C17—C13—C14—O3	−29.7 (2)
O5—C3—C4—C28	92.2 (3)	C12—C13—C14—O3	87.0 (2)
C2—C3—C4—C28	−82.1 (3)	C18—C13—C14—C15	−85.1 (2)
O5—C3—C4—C5	−144.7 (3)	C17—C13—C14—C15	36.8 (3)
C2—C3—C4—C5	41.0 (3)	C12—C13—C14—C15	153.5 (2)
C3—C4—C5—C6	175.64 (19)	C18—C13—C14—C8	70.8 (2)
C29—C4—C5—C6	55.3 (3)	C17—C13—C14—C8	−167.32 (18)
C28—C4—C5—C6	−66.5 (3)	C12—C13—C14—C8	−50.6 (2)
C3—C4—C5—C10	−52.7 (2)	C8—C14—C15—O3	101.0 (2)
C29—C4—C5—C10	−173.07 (19)	C13—C14—C15—O3	−104.6 (2)
C28—C4—C5—C10	65.1 (3)	O3—C14—C15—C16	105.2 (2)
C4—C5—C6—C7	−90.5 (2)	C8—C14—C15—C16	−153.8 (2)
C10—C5—C6—C7	136.50 (19)	C13—C14—C15—C16	0.6 (3)
C5—C6—C7—O6	−31.4 (3)	O3—C15—C16—O4	−132.8 (3)
C5—C6—C7—O7	151.5 (2)	C14—C15—C16—O4	158.6 (3)
C30—C8—C9—C11	127.1 (2)	O3—C15—C16—O2	48.4 (3)
C14—C8—C9—C11	−47.0 (2)	C14—C15—C16—O2	−20.2 (4)
C30—C8—C9—C10	−100.9 (3)	C14—C13—C17—O2	−58.0 (2)
C14—C8—C9—C10	84.9 (2)	C18—C13—C17—O2	61.6 (2)
C2—C1—C10—C19	118.2 (3)	C12—C13—C17—O2	−174.94 (18)
C2—C1—C10—C5	−6.1 (3)	C14—C13—C17—C20	−178.09 (18)
C2—C1—C10—C9	−123.0 (3)	C18—C13—C17—C20	−58.5 (3)
C6—C5—C10—C1	166.66 (19)	C12—C13—C17—C20	65.0 (2)
C4—C5—C10—C1	35.5 (2)	O2—C17—C20—C21	137.5 (3)
C6—C5—C10—C19	45.6 (3)	C13—C17—C20—C21	−99.2 (3)
C4—C5—C10—C19	−85.6 (2)	O2—C17—C20—C22	−44.4 (3)
C6—C5—C10—C9	−73.1 (2)	C13—C17—C20—C22	78.9 (3)
C4—C5—C10—C9	155.70 (18)	C22—C20—C21—O1	0.5 (3)
C8—C9—C10—C1	−81.7 (2)	C17—C20—C21—O1	179.0 (2)
C11—C9—C10—C1	46.8 (2)	C21—C20—C22—C23	−0.9 (3)
C8—C9—C10—C19	36.9 (3)	C17—C20—C22—C23	−179.3 (3)
C11—C9—C10—C19	165.38 (18)	C20—C22—C23—O1	0.9 (4)
C8—C9—C10—C5	158.90 (17)	C22—C23—O1—C21	−0.6 (4)
C11—C9—C10—C5	−72.6 (2)	C20—C21—O1—C23	0.0 (3)
C8—C9—C11—C12	48.3 (3)	O4—C16—O2—C17	178.2 (3)
C10—C9—C11—C12	−84.2 (2)	C15—C16—O2—C17	−3.0 (4)
C9—C11—C12—C13	−53.0 (3)	C20—C17—O2—C16	169.0 (2)
C11—C12—C13—C14	50.4 (2)	C13—C17—O2—C16	43.4 (3)
C11—C12—C13—C18	−69.3 (2)	C16—C15—O3—C14	−109.9 (2)
C11—C12—C13—C17	166.60 (19)	C8—C14—O3—C15	−114.1 (2)
C30—C8—C14—O3	99.9 (3)	C13—C14—O3—C15	107.6 (2)
C9—C8—C14—O3	−85.9 (2)	O6—C7—O7—C31	−0.4 (4)
C30—C8—C14—C15	32.5 (3)	C6—C7—O7—C31	176.7 (2)
